# The Cost of the Unit

**Published:** 1920-10-23

**Authors:** 


### The Hospital

The Workers' Newspaper of Administrative Medicine and Institutional Life, Administration, National Insurance and Health.

No, 1794, Vol. LXIX. SATURDAY, OCTOBER 23, 1920. PRICE TWOPENCE

### THE COST OF THE UNIT.

From the bewilderment and confusion caused by high prices as they affect wages, and wages as they affect high prices, at last emerges the solution—the reduction of the cost of the unit. That it is more to the profit and comfort of the employer, the employed, and the community generally to make and sell 5,000 articles at 15s. than 2,000 at a sovereign is now so plain as to become a commonplace recognised by most people. The whole of the difficulties in connection with the coal strike are centred round this very point.

That it is better to maintain one hundred beds in a hospital at an average price of £3 each rather than eighty at £3 9s. is the lesson underlying the facts and figures contained in the statistical report of King Edward's Hospital Fund for London for the year 1919 on the ordinary expenditure of 109 London hospitals. That there should have been a fall in the bed occupation during last year, in view of the discharge of military patients, is not surprising; indeed, many of the beds were extra to the ordinary establishment, having been placed in other parts of the hospitals not ordinarily used as wards, or in adjacent buildings. In 1918, therefore, there were 13,963 beds available, and these in 1919 were reduced to 12,589. The occupation in the two years respectively was 11,566 and 9,988. In 1918 the hospitals used eighty-three per cent. of the bed accommodation, but in 1919 only 79 per cent. Yet it may be said that practically the same overhead expenses were incurred, and that the general number of employees was not proportionately lessened.

It is a fault of the King's Fund statistical report that when cost per bed under such a heading as food is mentioned there is no indication as to the number of people who are being fed. We know, of course, the number of patients, but, beyond the amount paid in salaries and wages, we are given no clue as to the size of the staff—a condition, as shown by information provided in "Burdett's Hospitals and Charities," which differs surprisingly as between hospitals of the same character. Thus when we notice from the figures under review that in hospitals which received military patients the cost per bed under food has materially risen, while in hospitals for women it has remained almost stationary, it is inevitable that the question should arise as to whether the transition from war to peace conditions has happened without bed wastage.

Bed wastage at the present time is worse than a fault; it is a serious weapon in the hands of these who do not love the voluntary system, and who are waiting for an opportunity to attack it. Bed wastage spells waste of money, of effort, even more, of lives and of health.

Conclusions come to by Sir Napier Burnett in a recent address were that the present voluntary hospital provision for patients and staff is inadequate for existing requirements and that the present financial position of these voluntary hospitals is such as to put hospital extension entirely out of the question. This being so, it is all the more essential that what provision we have should be used to its utmost. There has been some talk of filling beds from a centre in each district in the same way that Service patients were sent to the military, Red Cross and auxiliary hospitals during the war. On this point we have thought it best to reserve our opinion, but at first sight it would appear that if there is one thing which would interfere more than another with the individuality of hospitals, or the keenness and interest of honorary medical staffs, it would be such a method. Most hospitals could not welcome the system, and their best argument against it would be to show a large occupational average. This can be done, as may be readily seen by an inspection of the useful tables in the Statistical Report of King Edward's Hospital Fund. To quote examples we may mention the Queen's Hospital for Children, with 112 beds and a daily average of in-patients of 109, and University College Hospital, with 322 beds and 308 of them occupied every day.

Much could be written about methods of allocating beds to patients, of "take-in" weeks, of certain beds set aside for certain physicians and surgeons, of groups of honorary staff for certain days, and of all the different plans made to suit different hospitals. Whether these plans be good or bad, economy in its highest meaning, the waiting-list long or short, the needs of the district, the present and future health of the community, and the many other considerations of value to the common weal, combine to call for a close and careful scrutiny of the systems for filling the beds in our voluntary hospitals. Then through resulting improvement, not necessarily with perceptibly more effort or appreciably more expenditure of money, we shall derive the last ounce of power from the hospital machine.

